# Supplementary material for: A Machine Learning Model for Predicting Unscheduled 72 h Return Visits to the Emergency Department by Patients with Abdominal Pain
Source: Diagnostics (Basel). 2021 Dec 30;12(1):82. doi: 10.3390/diagnostics12010082 (PMC8775134; doi:10.3390/diagnostics12010082)
Supplement: Supplementary file 1 [file diagnostics-12-00082-s001.zip › supplementary files/Supplementary File S1 Hyperparameters.pdf]

Logistic regression was implemented with python sklearn packages.
Hyperparameter was tuned with grid search algorithm with 10 fold stratified cross-validator 3 times. Evaluation metric is AUC (area under ROC curve).

Grids for tuning logistic regression
solvers = ['newton-cg', 'lbfgs', 'liblinear']
penalty = ['l2']
c_values = [100, 10, 1.0, 0.1, 0.01]

Random forest was implemented with python sklearn packages.
Hyperparameter was tuned with random search algorithm with 10 fold stratified cross-validator 3 times. Evaluation metric is AUC (area under ROC curve).

Grids for tuning random forest
```
rand_grid = {'bootstrap': [True, False],
    'max_depth': [10, 25, 50, 100],
    'max_features': ['sqrt','log2'],
    'min_samples_leaf': [1, 2, 4],
    'min_samples_split': [2, 5, 10],
    'n_estimators': [10, 100, 500]}
```

Extreme gradient boosting (XGB) was implemented with python sklearn packages (Scikit-Learn Wrapper API).
https://xgboost.readthedocs.io/en/latest/python/index.html

Hyperparameter was tuned with Hyperpot packages (Distributed Asynchronous Hyper-parameter Optimization) by using hyperopt fmin function (Tree Parzen Estimator).
http://hyperopt.github.io/hyperopt/

Evaluation metric is AUC (area under ROC curve).

Search space for Hyperpot
```
space={
'max_depth': hp.choice('max_depth', np.arange(3, 18, 1, dtype=int)),
'gamma': hp.uniform('gamma', 1,9),
'colsample_bytree' : hp.uniform('colsample_bytree', 0.5,1),
'subsample' : hp.uniform('subsample', 0.5,1),
'min_child_weight' : hp.choice('min_child_weight', np.arange(0, 10, 1, dtype=int)),
'eta' : hp.uniform('eta', 0.005, 0.3),
'n_estimators' : hp.choice('n_estimators', np.arange(20, 200, 5, dtype=int)),
'seed': 0,
'reg_alpha' : hp.choice('reg_alpha', np.arange(40, 180, 1, dtype=int)),
'reg_lambda' : hp.uniform('reg_lambda', 0, 1)
```
