# Supplementary material for: A Machine Learning Model for Predicting Unscheduled 72 h Return Visits to the Emergency Department by Patients with Abdominal Pain
Source: Diagnostics (Basel). 2021 Dec 30;12(1):82. doi: 10.3390/diagnostics12010082 (PMC8775134; doi:10.3390/diagnostics12010082)
Supplement: Supplementary file 1 [file diagnostics-12-00082-s001.zip › supplementary files/Supplementary File S2 Weights combinations of classifiers.pdf]

Importance of LR	Importance of RF	Importance of XGB	AUC of validation
1	1	1	0.711
2	2	1	0.712
1	1	2	0.707
1	2	2	0.709
2	1	1	0.712
2	1	2	0.710
**1**	**2**	**1**	**0.713**
1	1	1	0.711
1	6	1	0.710
1	6	1	0.710
1	1	6	0.696
1	6	6	0.702
6	1	1	0.707
6	1	6	0.709
6	6	1	0.711
1	10	1	0.708
1	1	10	0.692
1	10	10	0.700
10	1	1	0.704
10	1	10	0.709
10	10	1	0.711
1	14	1	0.706
1	1	14	0.690
1	14	14	0.699
14	1	1	0.703
14	1	14	0.709
14	14	1	0.711
1	18	1	0.705
1	1	18	0.688
1	18	18	0.699
18	1	1	0.703
18	1	18	0.709
18	18	1	0.710
