# Supplementary material for: A Machine Learning Model for Predicting Unscheduled 72 h Return Visits to the Emergency Department by Patients with Abdominal Pain
Source: Diagnostics (Basel). 2021 Dec 30;12(1):82. doi: 10.3390/diagnostics12010082 (PMC8775134; doi:10.3390/diagnostics12010082)
Supplement: Supplementary file 1 [file diagnostics-12-00082-s001.zip › supplementary files/Supplementary Table S1 Features site difference.pdf]

		Missing	Overall	Taipei branch	Linkou branch	p-Value
			N = 25151	N = 7723	N = 17428	
**Age**, mean(SD), years		0	46.8 (18.2)	46.4 (18.7)	46.9 (18.0)	0.057
**SEX**, n (%)	Male	0	10440 (41.5)	2760 (35.7)	7680 (44.1)	<0.001
**ER_visit_365**, mean (SD), times		0	1.4 (4.8)	1.6 (5.0)	1.3 (4.7)	<0.001
**Triage level**, n (%)	1	0	53 (0.2)	8 (0.1)	45 (0.3)	<0.001
	2		687 (2.7)	154 (2.0)	533 (3.1)	
	3		23097 (91.8)	6426 (83.2)	16671 (95.7)	
	4		1313 (5.2)	1135 (14.7)	178 (1.0)	
	5		1 (0.0)	0 (0.0)	1 (0.0)	
**ER_LOS**, mean (SD), hours		3	5.0 (11.4)	3.2 (7.3)	5.8 (12.8)	<0.001
**PULSE**, mean (SD), times		94	84.3 (15.8)	83.3 (15.6)	84.7 (15.9)	<0.001
**Last systolic pressure**, mean (SD)		399	134.2 (24.2)	131.7 (24.2)	135.3 (24.2)	<0.001
**Last diastolic pressure**, mean (SD)		408	80.3 (14.6)	74.9 (13.6)	82.7 (14.4)	<0.001
**Respiratory rate**,		185	17.9 (1.4)	17.5 (1.3)	18.0 (1.5)	<0.001

mean (SD)					
**X-ray**, n (%)	0	12987 (51.6)	3311 (42.9)	9676 (55.5)	<0.001
**Echo**, n (%)	0	520 (2.1)	37 (0.5)	483 (2.8)	<0.001
**CT**, n (%)	0	3375 (13.4)	732 (9.5)	2643 (15.2)	<0.001
