# Supplementary figures and images for: A Machine Learning Model for Predicting Unscheduled 72 h Return Visits to the Emergency Department by Patients with Abdominal Pain
Source: Diagnostics (Basel). 2021 Dec 30;12(1):82. doi: 10.3390/diagnostics12010082 (PMC8775134; doi:10.3390/diagnostics12010082)

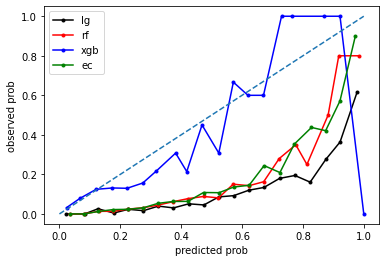

Supplement: Supplementary file 1 [file diagnostics-12-00082-s001.zip › supplementary files/Supplementary Figure S1 Calibration curves.png]

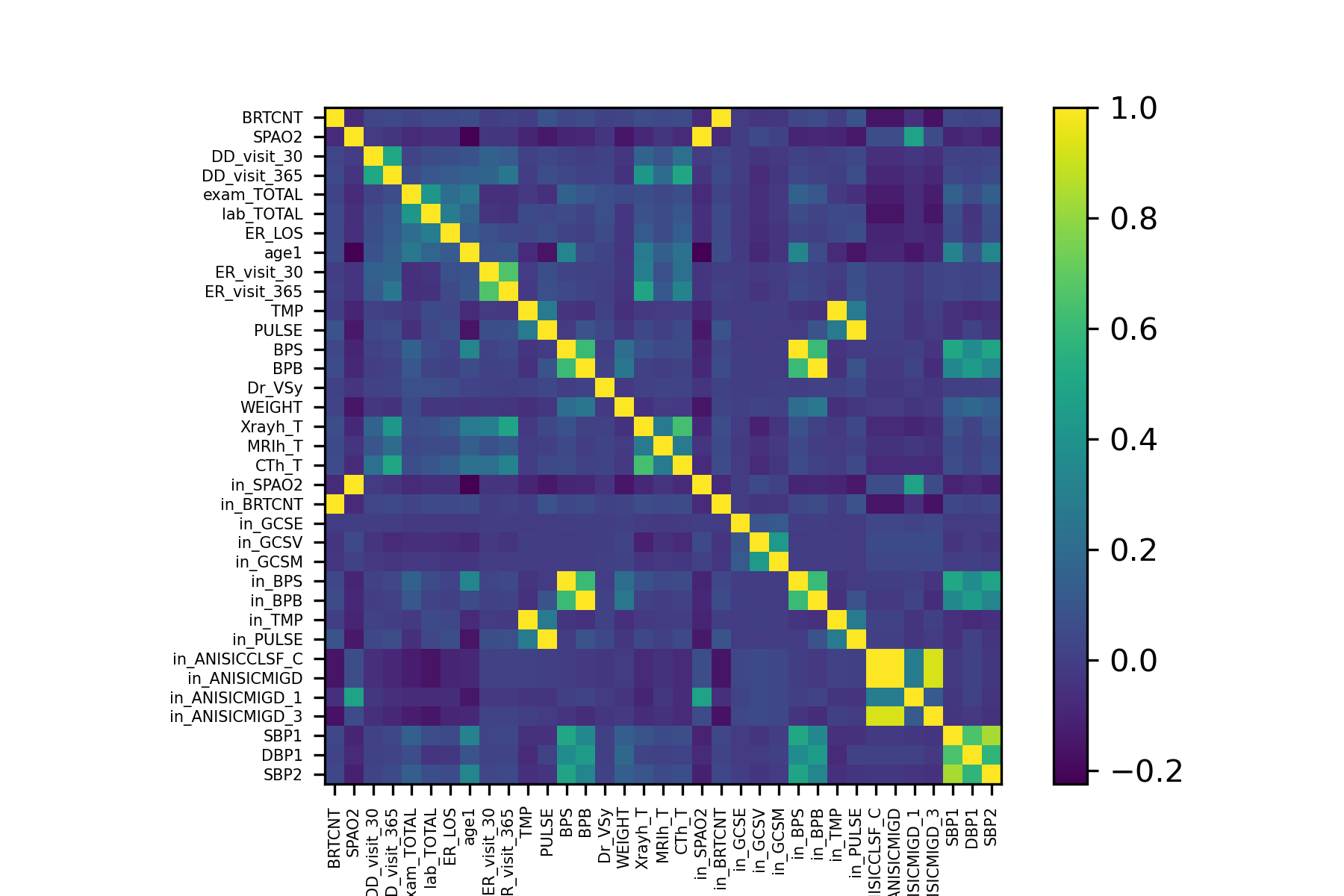

Supplement: Supplementary file 1 [file diagnostics-12-00082-s001.zip › supplementary files/Supplementary Figure S2 Continuous features correlation figure matrix.png]

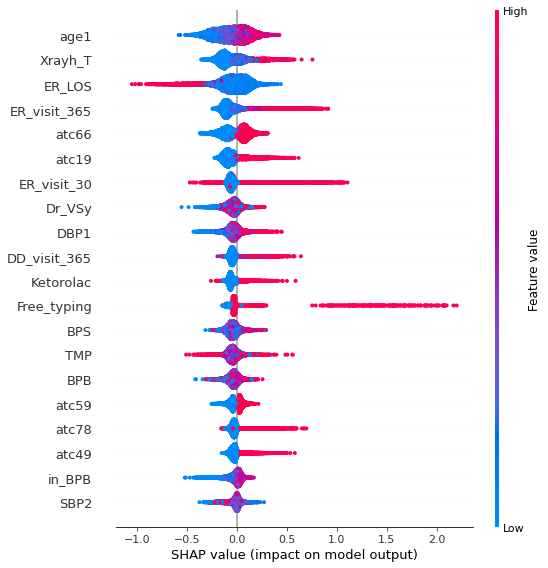

Supplement: Supplementary file 1 [file diagnostics-12-00082-s001.zip › supplementary files/Supplementary Figure S3 SHAP value of XGB.png]
